# Photoplethysmogram Biometric Authentication Using a 1D Siamese Network

**DOI:** 10.3390/s23104634

**Published:** 2023-05-10

**Authors:** Chae Lin Seok, Young Do Song, Byeong Seon An, Eui Chul Lee

**Affiliations:** 1 Department of AI & Informatics, Graduate School, Sangmyung University, Hongjimun 2-Gil 20, Jongno-Gu, Seoul 03016, Republic of Korea; 202231058@sangmyung.kr (C.L.S.); 202232031@sangmyung.kr (Y.D.S.);; 2 Department of Human-Centered Artificial Intelligence, Sangmyung University, Hongjimun 2-Gil 20, Jongno-Gu, Seoul 03016, Republic of Korea

**Keywords:** deep learning, photoplethysmogram, one-dimensional Siamese network, biometric, identification, lightweight

## Abstract

In the head-mounted display environment for experiencing metaverse or virtual reality, conventional input devices cannot be used, so a new type of nonintrusive and continuous biometric authentication technology is required. Since the wrist wearable device is equipped with a photoplethysmogram sensor, it is very suitable for use for nonintrusive and continuous biometric authentication purposes. In this study, we propose a one-dimensional Siamese network biometric identification model using a photoplethysmogram. To maintain the unique characteristics of each person and reduce noise in preprocessing, we adopted a multicycle averaging method without using a bandpass or low-pass filter. In addition, to verify the effectiveness of the multicycle averaging method, the number of cycles was changed and the results were compared. Genuine and impostor data were used to verify the biometric identification. We used the one-dimensional Siamese network to verify the similarity between the classes and found that the method with five overlapping cycles was the most effective. Tests were conducted on the overlapping data of five single-cycle signals and excellent identification results were observed, with an AUC score of 0.988 and an accuracy of 0.9723. Thus, the proposed biometric identification model is time-efficient and shows excellent security performance, even in devices with limited computational capabilities, such as wearable devices. Consequently, our proposed method has the following advantages compared with previous works. First, the effect of noise reduction and information preservation through multicycle averaging was experimentally verified by varying the number of photoplethysmogram cycles. Second, by analyzing authentication performance through genuine and impostor matching analysis based on a one-dimensional Siamese network, the accuracy that is not affected by the number of enrolled subjects was derived.

## 1. Introduction

As virtual reality and metaverse application areas expand, head-mounted displays (HMD) are being widely used to maximize immersion. However, since wearing an HMD makes it difficult to use conventional input devices, such as a keyboard or mouse, it is difficult to use conventional password or pattern drawing-based authentication methods [1]. Nonintrusive and continuous biometric authentication technologies can be considered natural and convenient authentication methods. However, face authentication is difficult to adopt as an occlusion of the HMD, and fingerprint or vein authentication cannot be considered because a separate sensor is required and the user’s cooperation is required. Iris or periocular biometrics can be considered, but a separate infrared imaging device must be added to the HMD [2]. The photoplethysmogram (PPG) is generally used to measure health indices such as heart rate or oxygen saturation and is a sensor that is included by default in even low-cost wrist wearable device types [3]. PPG can change its morphological characteristics according to human health, emotions, blood vessels, skeleton, and skin characteristics, and these characteristic changes can be considered as authentication means according to individual differences as well as individual health and emotional changes [4]. Biometric authentication is performed naturally without the user’s cooperation just by wearing a wrist wearable device, and it is very suitable for applying continuous authentication at regular time intervals while updating registered biometric information over time.

**Figure 1:**
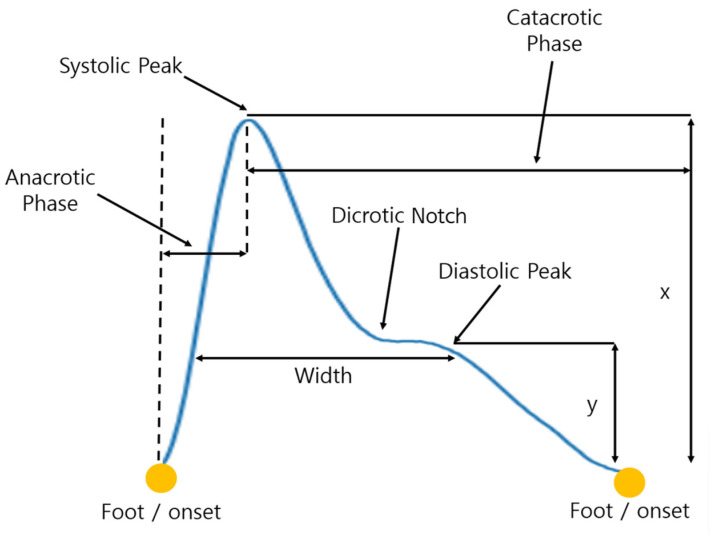
The waveform of a photoplethysmogram (PPG) signal.

PPG is a noninvasive technique for measuring the relative amount of blood in the skin. The principle of PPG signals is based on the brightness of the skin. Because hemoglobin is a light absorber, the brightness of the skin varies according to the blood volume caused by the heartbeat. The PPG signal is a measurement of the change in brightness due to the amount of blood [5]. In heart rate estimation, an electrocardiogram (ECG) requires two or three sensors to be attached around the heart, but PPG can monitor the heart rate in all parts of the body that can transmit light, such as earlobes, fingertips, and wrists. It is also widely used as an alternative to ECG to provide information on the pulse, simply by monitoring the changes in the intensity of the light transmitted from the body [6]. The PPG waveform is largely divided into two parts. The edge that first appears in the upward stroke of the systolic is called the anacrotic phase, and the edge of the diastolic leading to the fall is called the catacrotic phase. The dicrotic notch appears in the catacrotic phase, which is caused by the sudden closure of the aortic valve, resulting in retrograde blood flow and increased arterial blood volume in a short period [7]. Figure 1 shows the general PPG waveform and variables that reflect the movement of blood in blood vessels [8]. In addition, as the blood moves from the heart, it contains cardiovascular information, such as the condition of the heart and blood vessels, which can be captured using the PPG signals.

The various features of PPG signals enable biometric identification. Owing to the recent improvements in the functionality of wearable devices, wearable sensors using PPG have become popular, making it easy to acquire PPG signals from products such as the latest smartwatches and bands in a user-friendly manner [9]. In addition, because the signal is continuously acquired without the user’s conscious participation, it has the advantage of not causing inconvenience to the user during signal extraction. In this study, user identification was performed using PPG signals. Deep learning was used to distinguish between the PPG signals of different users, and after learning each PPG signal, the genuine and impostor identities were determined. PPG biometric identification, for example, self-identification through a smartwatch, can be useful in emergencies or situations involving inaction, noncooperation, or loss of consciousness by the patient [10].

The remainder of this paper is organized as follows. Section 2 discusses the existing PPG-based authentication methods. Section 3 presents the proposed deep learning model for authentication using PPG signals. The training of the model and its subsequent verification are discussed. Section 4 presents the detailed validation results. Finally, Section 5 presents the concluding remarks.

## 2. Related Works

The existing biometric-based methods using PPG include a feature extraction method, a machine learning model, and a method using a deep learning model.

### 2.1. Statistical Methods

Certain studies on biometric identification using PPG have extracted and used features from PPG without using machine learning or deep learning. Sancho et al. set a threshold for the PPG signal with noise reduction through high-pass filtering, and the upper and lower peaks were interpolated. The equal error rate (EER) was calculated by changing the cycle in four ways: the average of cycles, the Karhunen–Loève transform (KLT) of the average of cycles, multiple cycles, and the KLT of multiple cycles. The experimental results showed that the multiple-cycle method with 30 cycles had the lowest EER. However, it was difficult to continuously receive 30 cycles. In this study, the stability of the signal was maintained through the average of several segmentations to solve this problem [11].

### 2.2. Machine Learning-Based Approaches

Lee et al. segmented a PPG signal into single-cycle signals and then applied the average overlap to three single-cycle signals. Subsequently, the hybrid k-nearest neighbor (KNN)–random forest model was applied with the characteristics of the instantaneous frequency component through the Poincaré graph and the higher-order differential energy function, yielding 96% accuracy. However, in the above study, there is a problem in that the amount of computation and computation time is increased owing to the preprocessing for dividing the signal into single cycles and the complexity of the hybrid KNN–random forest model [12]. Karimian et al. created a PPG segment using a third-order Butterworth bandpass filter to remove noise from the PPG signal. Then, wavelet transform was applied to the PPG signal, the correlation characteristics were removed, dimensionality reduction was performed, and support vector machine (SVM), self-organizing map (SOM), and KNN were applied. The results showed an accuracy (ACC) of 91.46% ± 15.24 for SVM, 92.96% ± 15.44 for SOM, and 93.76% ± 15.59 for KNN. The sampling rate of the technique in recent wearable devices is approximately 30 Hz; however, in the above study, PPG signals extracted at 300 Hz were used, which is difficult to apply in real life. In addition, there are problems related to the increased amount of computation and low computation speed owing to the use of dynamic time warping and kernel principal component analysis in preprocessing [10].

### 2.3. Deep Learning-Based Approaches

Biswas et al. preprocessed PPG signals through a 1–18 Hz Butterworth filter, and heart rate and biometric identification were performed through two-layer 1D-CNN and a two-layer long short-term memory. In the structural performance category (SPC) dataset, the model showed a 5-fold cross-validation accuracy of 96% but the performance was comparatively very low (72% for the F1 score and 67% for precision) [13]. Luque et al. suggested that PPG could be utilized as a biometric indicator by using end-to-end learning with CNN. In this study, the PPG signal was segmented into 1 s units to include one cycle, and verification was performed using CNN. An average accuracy of 78.2% and an area under the curve (AUC) of 83.2% were achieved. However, this approach does not secure the safety of biometric identification [14]. Hwang et al. describe a method for biometric authentication using photoplethysmography (PPG) signals. A deep learning model that combines convolutional neural networks (CNN) and long short-term memory (LSTM) was applied to PPG signals, resulting in an average accuracy of 87.1%. However, this method is limited by the long inference time required in practice due to the high number of parameters and the complexity of the model. Additionally, preprocessing is performed using discrete wavelet transform (DWT) and zero padding, which may result in time complexity issues and potential signal corruption. In contrast, our proposed approach overcomes these limitations by preserving the unique characteristics of an individual’s PPG signals through the overlapping of multiple cycles. This method enables fast inference with a low number of parameters in a deep learning model, facilitating real-time use [15]. Zhao et al. devised a low-cost continuous system that utilizes the user’s pulse signal from a photoplethysmogram sensor in a wrist-worn wearable. It can be applied to nonclinical PPG measurements with motion artifacts (MA). We use the MA filtering method to mitigate motion noise. We also use the gradient boosting tree (GBT) method to identify common fiducial features and develop an adaptive classifier. An experiment using a wrist-worn PPG detection platform with 20 participants shows that our system can achieve a high CA accuracy of over 90% and a low false positive rate of 4% when detecting random attacks. The previous paper, like this study, studies effortless continuous authentication using PPG signals. However, in order to make a quality signal, the entire signal is used by correcting the signal using MA, whereas in this paper, only a single signal with quality is used for authentication. Authentication can be performed more securely by excluding false signals due to noise [16].

The improvement of this study is as follows. First, reduce noise while maintaining features in PPG data through the multicycle averaging method. Second, the Siamese network, a 1D-CNN, reduces feature extraction and time with fewer parameters.

## 3. Methods

### 3.1. Dataset

**Figure 2:**
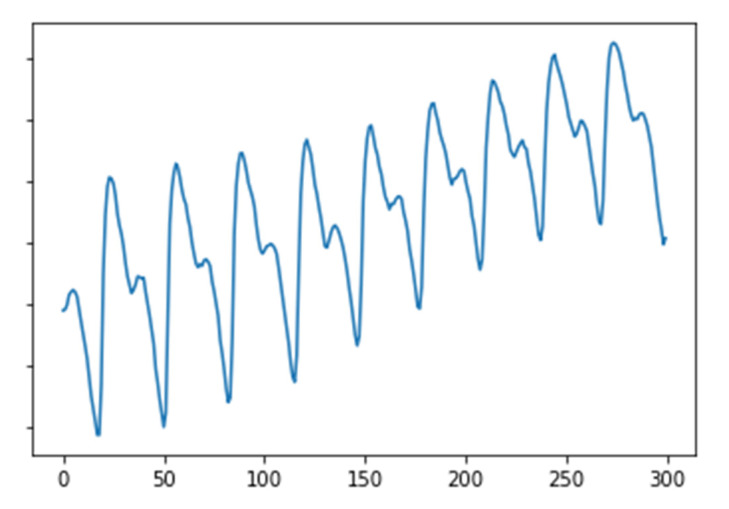
Example of PPG data.

The proposed model was evaluated on the publicly available Real-World PPG dataset [17,18]. The signals were measured under uncontrolled conditions. The dataset consisted of PPG signals from 35 healthy persons, and the signals were recorded for 6 s at a sampling rate of 50 samples/s. Figure 2 shows an example of the PPG data. The dataset contains a total of 2074 data of which 1374 (approximately 66%) and 700 (approximately 34%) were used as the training and test sets, respectively. The PPG signals that exist in the Real-World PPG dataset constitute a low-noise dataset. This study utilized the dataset to reduce the impact of noise, such as motion artifacts, and investigate the potential of PPG waveform characteristics to serve as biometric identifiers.

### 3.2. Data Processing

**Figure 3:**
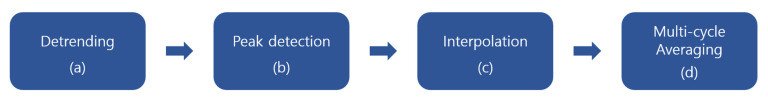
Preprocessing steps.

A PPG signal is a noisy signal caused by external environmental elements, such as motion or power line interference [19]. Signal preprocessing was performed to increase the accuracy by minimizing the effect of such noise on the system performance. A bandpass filter or low-pass filter is generally used for preprocessing the PPG signal, but such a filter was not used in this study because the unique characteristics of each person are available in the high-frequency region. In this study, a single-period signal was extracted from the original signal with high-frequency components, and a method of overlapping and averaging the single-period signal was proposed to reduce external noise and maintain the characteristics of each person. The proposed preprocessing method is divided into four steps, as shown in Figure 3.

#### 3.2.1. Detrending

**Figure 4:**
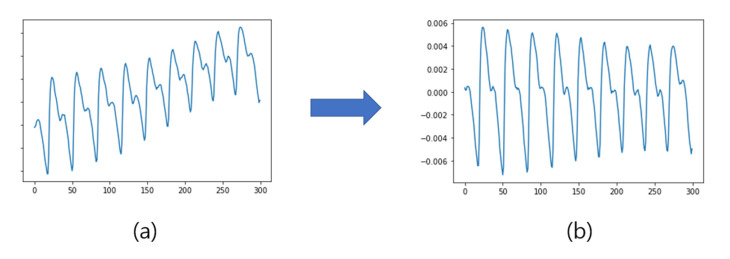
Results of detrending: (**a**) original signal; and (**b**) processing result.

In this study, before calculating the average by overlapping the single cycles, it was essential to accurately find the peak points and proceed with segmentation into single cycles. A detrending method was used for this purpose. Detrending is a method of removing linear data trends. Signals that pass through detrending have reduced overall fluctuations and can accurately exhibit the peaks [20]. In addition, normalization is performed on the signal that has passed through detrending. Because of this, the average of the signal becomes zero, the information on the amplitude can be maintained, and the signals can be overlapped stably. In general, detrending is the process of subtracting the line of best fit from the mean or least squares of the data. In this study, the signal was detrended by subtracting the moving average computed with a moving window, followed by normalization. The formula for computing the moving average within a window is given in Equation (1), and the formula for detrending and normalization of the signal using Equation (1) is given in Equation (2). Figure 4 shows the results of applying these formulas to the signal. The length in Equations (1) and (2) means the length of the window.

(1) $${\mathrm{mean}}_{i}=\frac{\sum_{j=i}^{i+\mathrm{win}\_\mathrm{size}}{\mathrm{signal}}_{j}}{\sum_{j=i}^{i+\mathrm{win}\_\mathrm{size}}1},\;\;i=1,\cdots ,\mathrm{length}$$

(2) $${\mathrm{detrended}\_\mathrm{signal}}_{i}=\frac{{\mathrm{signal}}_{i}-{\mathrm{mean}}_{i}}{{\mathrm{mean}}_{i}},\;\;i=1,\cdots ,\mathrm{length}$$

#### 3.2.2. Peak Detection

**Figure 5:**
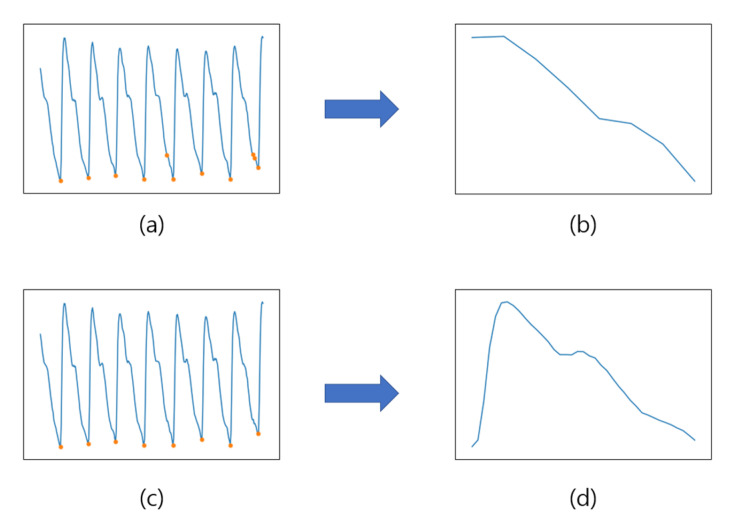
Results of peak detection (blue line: PPG signal): (**a**) result of finding the foot points (orange circles) in the PPG signal without distance; (**b**) result of single-cycle extraction without distance; (**c**) result of finding the foot points (orange circles) in the PPG signal using distance; and (**d**) result of single-cycle extraction using distance.

Because the average of the signal that has gone through detrending is 0, we find a value less than 0 among the inflection points and set it as a peak. In this study, the inflection point owing to noise can be found as a peak by using the original signal that has not passed through the filter. (a) and (b) in Figure 5 show the case of finding the inflection point owing to noise as a peak. To solve the problem due to these false peaks, exception processing is performed by ignoring peaks that determine an interval shorter than the threshold, which is set based on the minimum heart rate. Subsequently, segmentation is performed between two successive foot points to obtain a single-cycle signal. (c) and (d) in Figure 5 show the result of segmentation into a single-cycle signal using the correct foot point in the PPG signal.

#### 3.2.3. Interpolation

**Figure 6:**
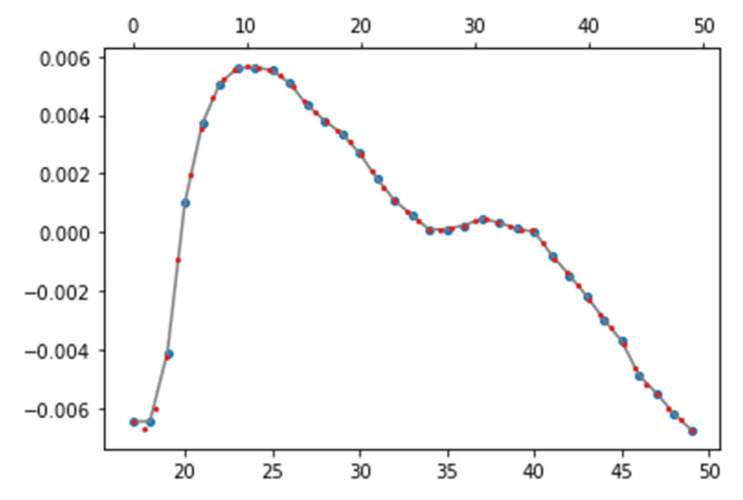
Results of interpolation (blue dots: original signal; gray line: a line connecting the dots corresponding to the original signal; red dots: interpolation results).

The length of each cycle is different because each signal has a different heart rate. If the length of the cycle is different, 50 points at equal intervals are extracted based on the time axis because the average value cannot be obtained at the same point. Interpolation was used to obtain equal intervals. If the interpolation method proceeds linearly, the PPG signal with a curve cannot be interpolated accurately. Therefore, quadratic spline interpolation is used, which employs a low-degree quadratic equation by dividing the entire signal into subsections [21]. The quadratic spline interpolation method follows Equation (3), and Figure 6 shows the result of the formula.

(3) $$S_{i}=y_{i}+Z_{i}\left(x-x_{i}\right)+\frac{Z_{i+1}-Z_{i}\;}{2(x_{i+1}-x_{i)}}\left(x-x_{i}\right){\;}^{2}$$

#### 3.2.4. Multicycle Averaging

The unique characteristics of each person appear in the high-frequency region. In this study, to reduce noise while maintaining the high-frequency region, a method of calculating the average by overlapping several unfiltered single-cycle signals was used. This reduces noise, such as motion noise included in the signal, and maintains the cardiovascular characteristics of each person. This is because the noise, such as motion noise, is not regular within a single-cycle signal, but human cardiovascular characteristics appear at a common location within one cycle signal. The formula for converting several single cycles into a single cycle is shown in Equation (4).

(4) $$X=\frac{\sum_{k=1}^{N}x\left(k\right)}{N}$$

**Figure 7:**
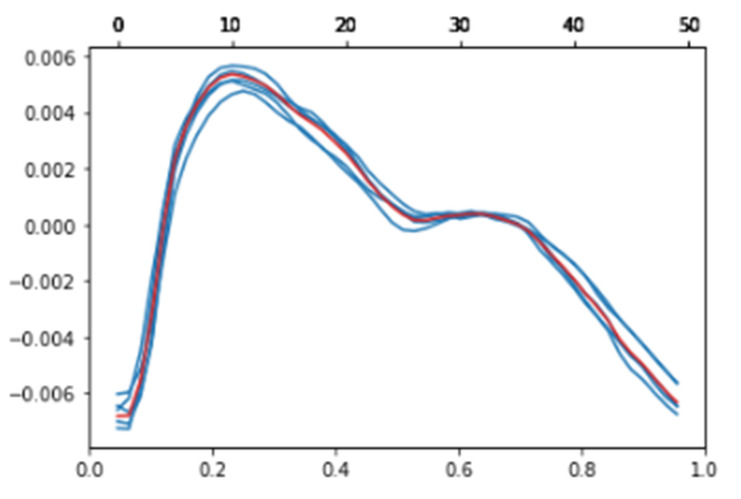
Multicycle averaging results (Blue lines: *N* single cycle signals. Red line: the averaged one) (*N* = 5).

*X* denotes a signal created through the formula and *x* denotes a single cycle extracted from the original signal. *N* denotes the number of single cycles that should be overlapped. Figure 7 shows the results of multicycle averaging.

**Figure 8:**
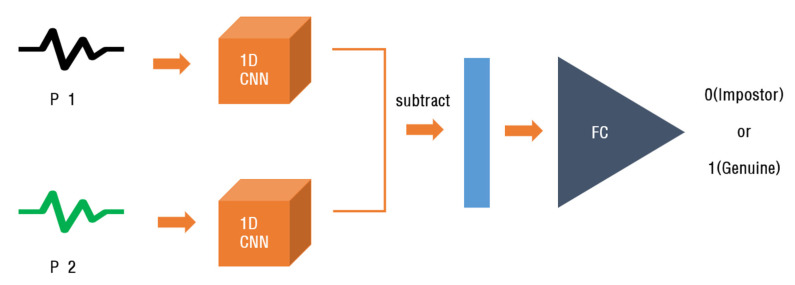
Structure of proposed Siamese network using 1D-CNN.

In Figure 8, the blue line is the overlapping of *N* single-period signals, and the red line shows the result of the formula. In this study, to prove that the multicycle averaging method is effective, one to five single cycles were overlapped and the biometric results were compared.

### 3.3. Model

The proposed model is a Siamese network with a 1D-CNN and uses the similarity between two signals to distinguish between genuine data and impostor data. In the case of biometrics, it is recommended to secure a large amount of initial data. However, collecting a large amount of data is challenging as it causes inconvenience to users. Therefore, when data for a specific class cannot be obtained in large quantities, the similarity is measured using a Siamese network, which is a one-shot learning-based network designed to classify the class.

A Siamese network is a type of neural network that consists of two or more identical neural networks that share the same weights. The network compares two inputs and generates an output vector that represents their similarity. Siamese networks are used for processing and comparing high-dimensional or differently-shaped data. The typical structure of a Siamese network consists of two convolutional neural networks (CNNs), where each input image is fed into each CNN. Each layer of the CNN extracts features from the input, and the output of each layer becomes the input of the next layer. When two CNNs process each input image, two vectors are generated. These vectors represent the features of each input image, and their similarity can be calculated by measuring the similarity of the vectors. For example, in the field of face recognition, a Siamese network receives two face images as inputs and calculates the similarity between them to improve the accuracy of face recognition. In addition, in the field of image search, Siamese networks are used to measure the similarity between the input image and the search target image to find the most similar image [22].

Performance evaluation of traditional biometrics is performed based on the Bayesian minimum error decision theory through genuine/impostor matching. However, since previous deep learning models are focused on the role of creating feature vectors, performance evaluation is naturally approached as a clustering problem of subjects. Since the Siamese network has a structure suitable for discriminatingly learning the difference or similarity by receiving data of the same structure as a pair, it is suitable for performance evaluation of traditional biometrics. Therefore, in this study, in order to compare a pair of PPG cycles, we chose the Siamese network structure, as shown in Figure 8.

### 3.4. Model Structure

**Figure 9:**
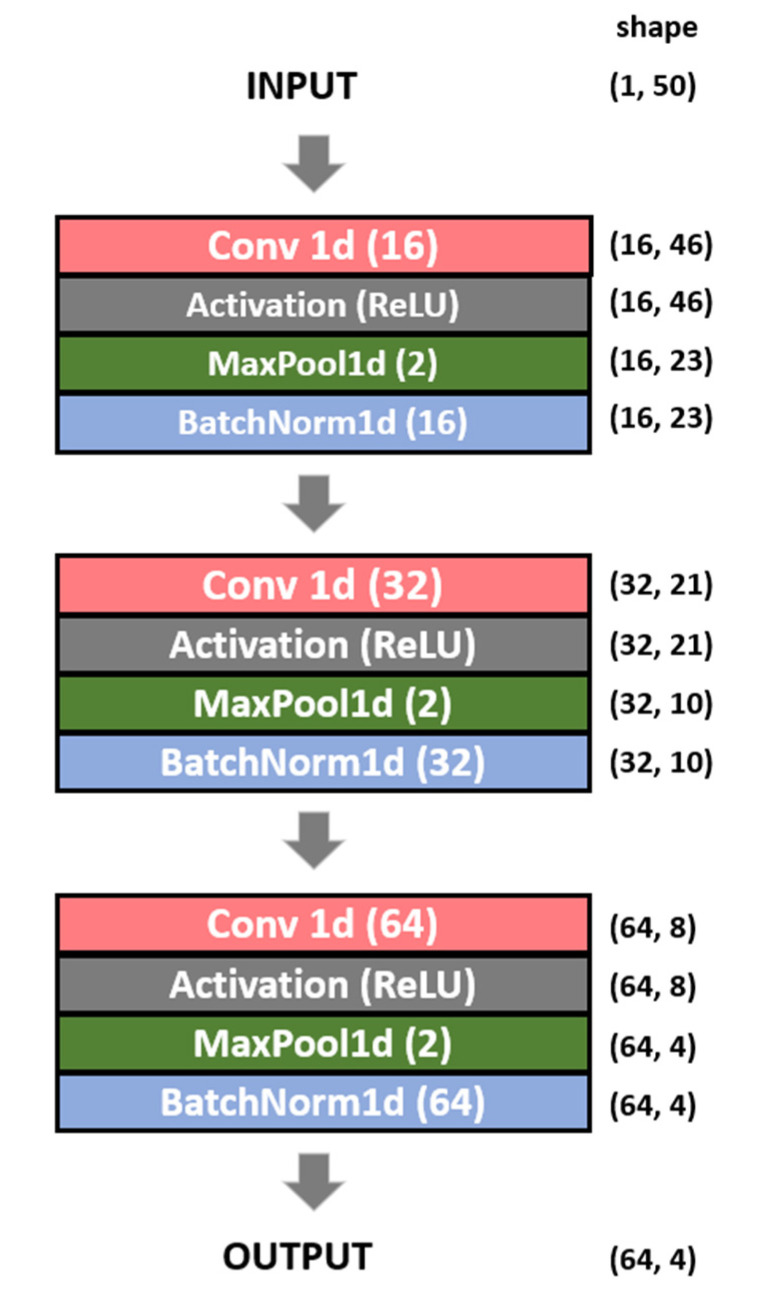
Structure of the twin network.

The structure of the proposed 1D-CNN Siamese network model can be divided into two parts. The first is a twin network that generates feature vectors from two signals, and the second is a fully connected (FC) layer that calculates the similarity between two feature vectors. A twin network consists of a sequence of convolution and pooling layers that extracts features from a signal. It comprises three convolution layers. Each layer involves convolution, max pooling, and batch normalization, and each layer has the same filter size. The input data are compressed into a feature vector that accurately represents the signal by passing it through the network. Figure 9 shows the structure of the twin network.

**Figure 10:**
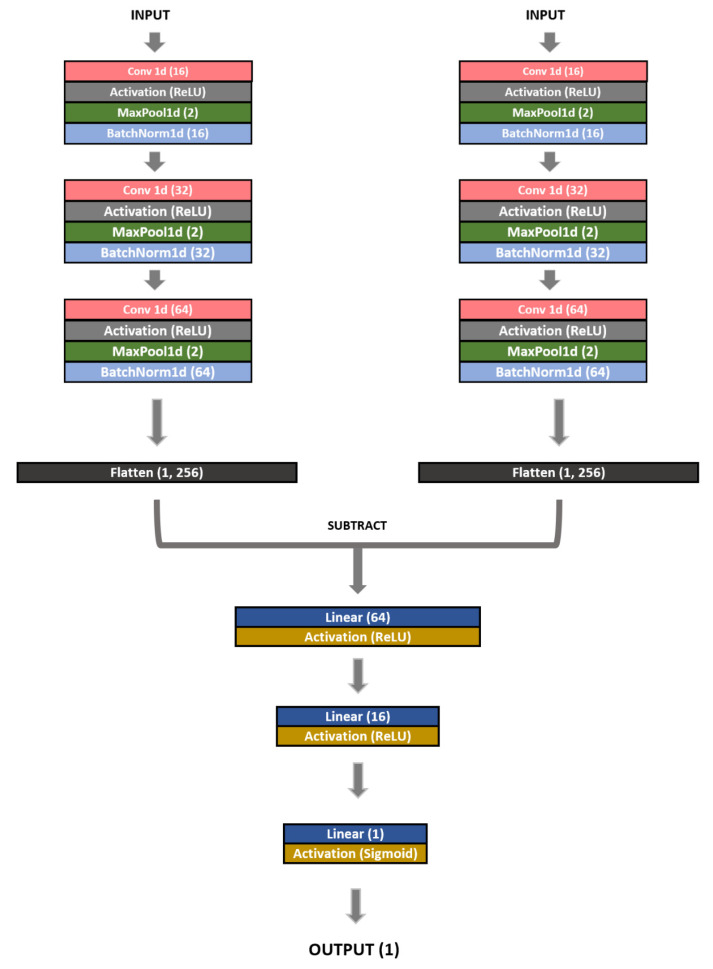
Overall structure of the model.

The difference between the two feature vectors extracted through the twin network is calculated and converted into one vector. The twin network result is passed through the FC layer and used to calculate the similarity between the two signals to be compared. The last of the FC layers applies a sigmoid function and outputs values between 0 and 1. This is the probability that the input and reference images will be the same. Figure 10 shows the overall structure of the model.

### 3.5. Training

The proposed model receives two signals as inputs. The model learns randomly by using genuine and impostor data. However, the number of impostor data points was greater than the number of genuine data points, causing the model to be biased toward the impostor data. Therefore, samples of genuine data and impostor data were selected and inputted with a probability of 50% so that the model was not biased toward a specific type of data. A total of 1374 training datasets were learned per epoch; thus, 687,000 datasets were learned in 500 epochs.

Binary cross-entropy loss (BCE loss), which is frequently used in binary classification problems, was used as the loss function [23]. The formula for calculating the cross-entropy between the actual label and the predicted value is given in Equation (5).

(5) $$BCELoss\left(\hat{y},y\right)=-(y\times \log\hat{y}+\left(1-y\right)\times \log\left(1-\hat{y}\right))$$

This study was conducted in the PyTorch environment, and the model was trained and tested on a laptop with an i7 CPU and 16 GB of memory. The Adam optimizer was used for learning to find the global minimum [24], and the learning rate was set to 0.0001 because it was difficult to find the minimum if the loss fluctuated significantly. The batch size was set to 32 and the number of training epochs was set to 500. The learning proceeded without early stopping.

## 4. Results

**Figure 11:**
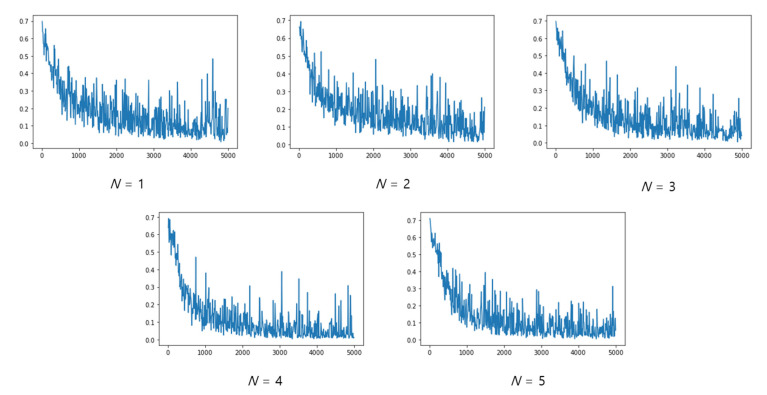
Training loss graph according to *N*.

**Figure 12:**
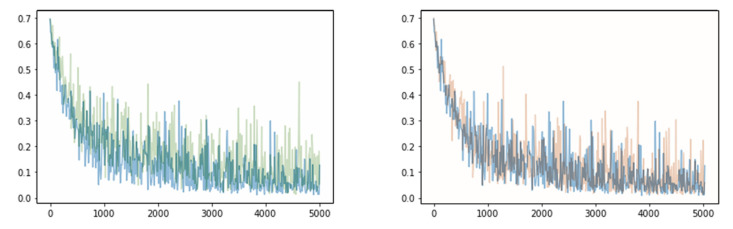
Comparison of loss (green: *N* = 1; orange: *N* = 4; and blue: *N* = 5).

Model tests were performed on the entire test dataset, that is, 244,650 data points. At this time, the number of genuine data points was 6650 (=_20_C_2_ × 35 subjects) and the number of impostor data points was 238,000 (=_700_C_2_ − the number of genuine pairs). First, to evaluate the performance according to the number of overlapping single cycles, learning was conducted by increasing the value of *N* in Equation (1) from 1 to 5. Because the input was provided randomly during the training, the range of change in the loss appears to be large. As shown in Figure 11, as the value of *N* increases (as the number of overlapping cycles increases), the range of change in the loss value decreases and the learning proceeds stably. Figure 12 depicts a graph of the loss when *N* is 1, and the loss when *N* is 4 overlaps with the loss when *N* is 5, which confirms that the loss decreases more stably as *N* increases.

In addition, as the training proceeded stably, the accuracy of the biometric gradually increased. This can be confirmed by the data in Table 1. The difference in accuracy between the case where only a single cycle is used and that where five single cycles are overlapped is approximately 5%.

**Figure 13:**
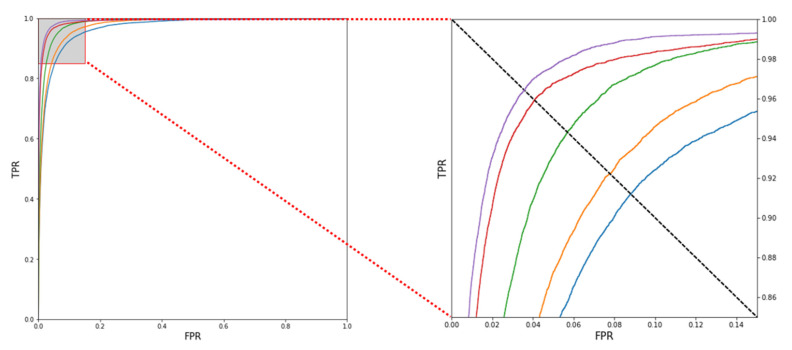
Receiver operating characteristic (ROC) curve according to N value (purple line: *N* = 5; red line: *N* = 4; green line: *N* = 3; orange line: *N* = 2; and blue line: *N* = 1).

The receiver operating characteristic (ROC) curve shows the performance of a binary classifier for various thresholds, and the upper-left curve indicates a better classifier [25]. Figure 13 shows the ROC curve drawn using the false positive rate (FPR) and true positive rate (TPR), and the performance was confirmed by enlarging the axis. As N increases, the curve approaches the upper-left corner, which means that the performance of the binary classifier improves.

The AUC score indicates the area under the ROC curve. The closer the AUC score is to 1, the better the classification performance of the model; the closer the AUC score is to 0.5, the poorer the classification ability of the model. Table 2 shows the AUC scores for various *N* values; the AUC score increases as the *N* value increases. This indicates that the classification performance of the model increases as the *N* value increases.

**Figure 14:**
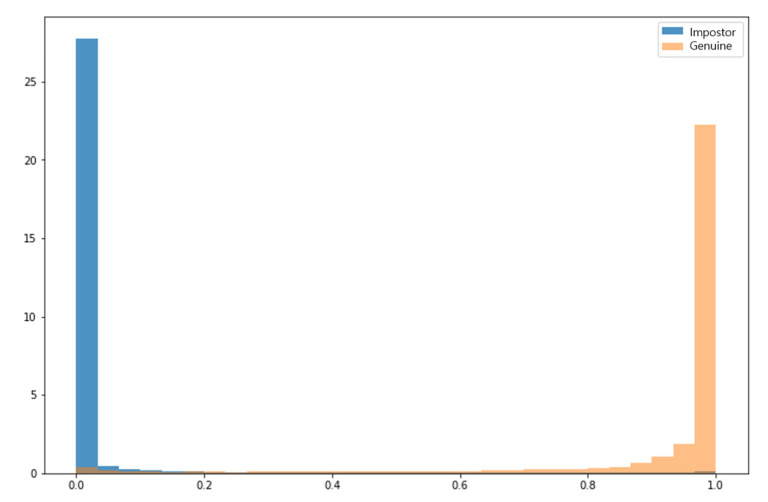
Genuine–impostor histogram (blue bar: impostor; and orange bar: genuine).

The above results confirm that the multicycle averaging method maintains the unique characteristics of each person contained in the signal while reducing the signal noise that deteriorates the model learning. In this study, five single cycles were overlapped to test the proposed model. The performance evaluation used the genuine–impostor distribution graph, that is, EER, which is a traditional biometric index and was conducted with the same 244,650 test data points used previously. Figure 14 shows the result of drawing the genuine–impostor distribution using the similarity values of the genuine and impostor combinations. A value of 1 indicates that the two signals are very similar, and 0 indicates that they are different signals. Figure 14 shows that the value for the impostor data is generally close to 0, whereas that for the genuine data is generally close to 1. This indicates that the model exhibits excellent performance in determining the similarity between the signals.

**Figure 15:**
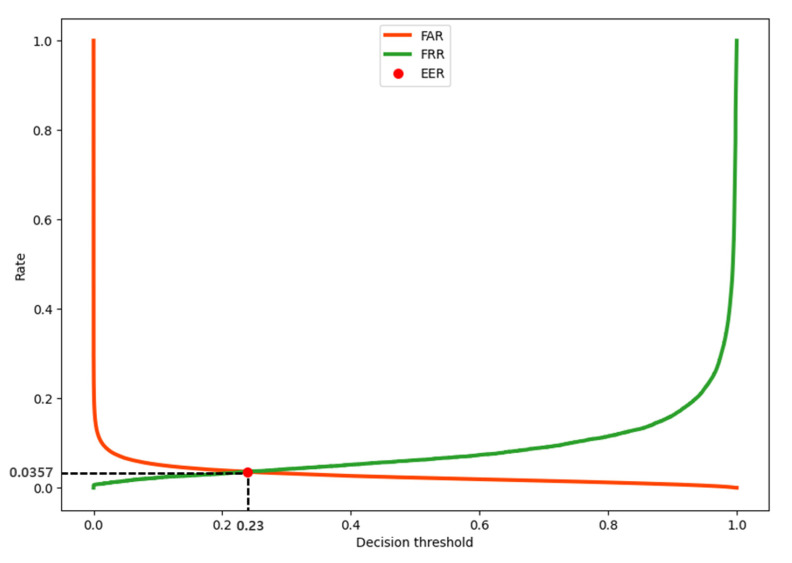
FAR–FRR trend as the change in decision threshold and EER.

The EER is an indicator of biometric performance. It refers to the point at which the false acceptance rate (FAR) and false rejection rate (FRR) are the same; the lower the EER, the better the biometric identification. Here, FAR is the probability of identifying the wrong biometric information, and FRR is the probability of failure to identify one’s biometric information [26]. Figure 15 shows the FAR, FRR, and EER. In this study, the EER was 0.0357. In addition, when the FAR, which is an important value in biometrics, is close to zero, the FRR is approximately 0.2.

## 5. Conclusions

In this study, we proposed a biometric identification method using PPG and a 1D-CNN Siamese network. Existing methods use filters to remove noise from PPG data; however, since a person’s unique characteristics can appear in the high-frequency range, the multicycle averaging method was used to reduce noise while maintaining information about the characteristics. The multicycle averaging method was used to reduce noise in the PPG data while maintaining the characteristics of each person. In addition, to verify the effectiveness of the multicycle averaging method, the loss, accuracy, ROC curve, and AUC score were compared while increasing the value of *N* from 1 to 5. It was proven that the multicycle averaging method is effective for all indicators. However, although the accuracy increases as multiple single-cycle signals are overlapped, the identification time also increases. Hence, biometric identification was performed using data of approximately 5 to 8 s, including up to five single-cycle signals, for a faster identification time. The authenticity of the signals that underwent multicycle averaging was determined using the proposed 1D-CNN Siamese network. Features were extracted from the signal through the twin network of the model by using a small number of parameters and data, and the difference between the extracted feature vectors was used to precisely calculate the similarity while passing through the FC layer. The similarity extracted through the Siamese network is a value between 0 and 1, so the results can be analyzed using various indicators used in traditional biometric recognition. This study showed excellent results for several biometric indicators (genuine–impostor distribution graph, ROC curve, AUC score, and EER). In this paper, we analyzed whether the characteristics of PPG can be used as biometric identifiers using the Real-World PPG dataset, which is a low-noise dataset. The results showed that the features of PPG are capable of distinguishing individuals and have the potential as biometric indicators. However, since we used a low-noise dataset, the performance may significantly deteriorate when using noisy data. Therefore, in future studies, we aim to refine the data from signals that contain noise and utilize them for biometric identification.

To implement cyber security in virtual reality and metaverse applications, rigorous and thorough work is required. Biometric authentication technology is recognized as a valid alternative to the common ID and password-based access methods in user authentication. In this study, we used PPG to achieve unobtrusive and continuous biometric authentication while demonstrating good results. However, it is important to note that in addition to the development of user authentication models, continuous attention, and improvement in all areas of cyber security are necessary. This includes addressing vulnerabilities in communication channels and processing algorithms found in biometric readers, smart card readers, and network-connected workstations, among others [27]. Therefore, ongoing efforts toward cyber security are crucial for ensuring the safety and protection of sensitive information in virtual reality and metaverse applications.

## Figures and Tables

**Table 1:** Accuracy according to *N*.

*N*	1	2	3	4	5
Accuracy	92.64	92.42	95.13	96.36	97.23

**Table 2:** AUC (Area Under Curve) score for various *N* values.

*N*	1	2	3	4	5
AUC	0.967	0.974	0.984	0.988	0.990

## Data Availability

Since the data used in this study is a public open dataset, it can be used by contacting the data holder.
